# Insulinoma masquerading as a loss of consciousness in a teenage girl: case report and literature review

**DOI:** 10.1186/s13633-017-0049-7

**Published:** 2017-10-17

**Authors:** Meghana Gudala, Mahmuda Ahmed, Rushika Conroy, Ksenia Tonyushkina

**Affiliations:** Division of Pediatric Endocrinology, Baystate Children’s Hospital, 50 Wason Ave, Springfield, MA 01199 USA

**Keywords:** Hypoglycemia, Insulinoma, Adolescents

## Abstract

**Background:**

Hypoglycemia due to a pancreatic beta cell neoplasm - insulinoma, is uncommon with only a few cases described. We report on a previously healthy 15-year-old Hispanic female with insulinoma who presented with a loss of consciousness due to hypoglycemia unawareness.

**Case presentation:**

EM was first brought to the emergency department (ED) after she was found unresponsive at home with point of care (POC) glucose of 29 mg/dL(1.6 mmol/L) documented by emergency medical services (EMS) upon arrival. After treatment with dextrose and normal laboratory evaluation, including complete blood count, basal metabolic profile and urine drug screen, she was sent home with recommendations to follow-up the next day with an endocrinologist. Due to insurance issues, the family did not keep the appointment. Two days later, she returned to the ED with POC of 19 mg/dL (1.05 mmol/L). Detailed history review identified vague fatigue, excessive sleepiness, poor oral intake and weight gain for a 2–3 month period and no suspicion for drug, alcohol or prescription medication abuse. Family history of multiple endocrine neoplasia was negative. Physical examination revealed mild acanthosis nigricans and a body mass index of 32.8 kg/m^2^ (98th percentile). Laboratory evaluation showed elevated insulin with low cortisol and growth hormone levels at the time of hypoglycemia. Abdominal magnetic resonance imaging revealed a pancreatic mass, also supported by ultrasound, computed tomography and positron emission tomography scans. The patient underwent a partial pancreatectomy with removal of a well-circumscribed insulinoma from the anterior-superior aspect of the pancreatic neck confirmed by histology. Hypoglycemia resolved post-operatively and she remained euglycemic during a 48-h cure fast. At her 3-month follow-up visit, she had no symptoms of hypoglycemia.

**Conclusion:**

Documented hypoglycemia in an otherwise healthy adolescent should be fully investigated before discharging a patient. Even a short duration of symptoms should prompt, in-depth diagnostic evaluations to rule out a potentially life threatening diagnosis of insulinoma.

## Background

Hypoglycemia, defined as a serum glucose level below 55 mg/dl (3 mmol/L), [1–3] is uncommon beyond infancy and early childhood in individuals without diabetes mellitus [4]. A retrospective pediatric study reported that hypoglycemia accounted for 0.034% of total emergency department (ED) visits [4]. While congenital hyperinsulinism and ketotic hypoglycemia are common causes of hypoglycemia in infants and children respectively [4], typical causes in adolescents include the surreptitious use of insulin, insulin secretagogues, alcohol [1], and medications [5]. Other causes of hypoglycemia are deficiency in adrenal and growth hormones, late presentation of congenital hyperinsulinism, glycogen storage diseases, disorders of gluconeogenesis, defects of fatty acid oxidation, insulinomas [1] and non-islet cell tumors [6], dumping syndrome seen post gastric-bypass surgery [7] and renal failure and sepsis in critically ill patients [1]. Evaluation and management of hypoglycemia is indicated only in patients with documented Whipple’s triad, which includes symptoms of hypoglycemia, a documented low plasma glucose level and a history of resolution of symptoms after an increase in plasma glucose level [1].

Insulinoma, a pancreatic tumor, is a rare cause of hypoglycemia. It has an approximate incidence of 1 in 250,000 patient-years with a median age of surgical diagnosis at 47 years. It occurs in all ethnic groups, at all ages, and is slightly predominant in women. Insulinoma is mostly a single benign tumor; only less than 10% of cases are malignant, presenting as multiple tumors or associated with Multiple Endocrine Neoplasia Type 1 (MEN 1) [8]. They are rare in children and adolescents with only a few cases reported worldwide [8–30] (Table 1).

**Table 1:** Previously published case reports of insulinoma in children (1960 - present)

Author	No. of cases	Age –years (Diagnosis/surgery)	Gender	Symptoms	Symptom duration (months)	Imaging/Tumor size(cms)
Present case	1	15	F	Loss of consciousness, vague fatigue, excessive sleepiness, jitteriness, tremors and sweating	2–3	MRI/1.5
Cameroglu et al. (2016) [9]	1	16	M	Shakiness, dizziness, disorientation, and unsteadiness	4	MRI/9.6
Halpin et al. (2016) [10]	1	15	F	Fatigue, confusion, poor concentration, irritability and “staring off”	8	MRI/1.3
Mirion et al. (2016) [11]	1	11	M	Diffuse abdominal pain, cold sweats, confusion, tremor, paresthesias	5	MRI/1.0
Bhatti et al. (2016) [12]	12	4–16	M (7) F (5)	NA	4–108	NA/0.7–2.0
Jung et al. (2015) [13]	1	11	F	Seizures, palpitations, sweating	5	MRI/3.0
Padidela et al. (2014) [14]	9	3–15	M(4) F (5)	Seizures (3)	1–24	MRI (7) CT (2)/0.8–2.0
Ahmed et al. (2014) [15]	1	10	M	Episodic tremulousness, diaphoresis, increased hunger, confusion and fainting	1	CT/1.7
Jyotsna et al. (2014) [16]	1	14	M	Loss of consciousness, seizures	24	MRI/1.8
Peranteau et al. (2013) [17]	8	4–26	M (6) F (2)	NA	1–84	MRI (3) CT (2) ^18F^DOPA PET (3)/0.7–1.8
Kao et al. (2013) [18]	1	9	F	Seizures, early morning behavioral changes	4	CT/1.4
Horvath et al. (2013) [19]	1	16	M	Seizures, unusual mental behavior	6	CT/1.6
Ide et al. (2012) [20]	1	13	M	Seizures	Unknown	CT/1.9
Blasetti et al. (2011) [21]	1	17	F	Seizures	9	CT/1.5
Janem et al. (2010) [22]	1	12	M	Abdominal pain, generalized weakness, sweating and drowsiness	4	MRI/NA
Ozen et al. (2009) [23]	1	16	M	Seizures, syncope, fatigue, weakness and somnolence	12	CT/0.8
Strong et al. (2007) [24]	1	13	M	Seizures	1	MRI/1.8
Jaladayan et al. (2007) [25]	1	13	F	Seizures, confusion, unresponsiveness, psychomotor slowing	6	MRI/2.5-first tumor – 0.7-0.9- second tumor
Jaksic et al. (1992) [26]	2	8	M (1) F (1)	NA	NA	A/1
Service et al. (1991) [8]	13	NA	NA	NA	NA	NA/NA
Grosfeld et al. (1990) [27]	5	NA	NA	Seizures (1)	NA	CT (3) U/S (1)/NA
Wolfsdorf et al. (1979) [28]	1	8	F	Loss of consciousness, lethargy	0.5	NA/1.1
Mann et al. (1969) [29]	5	3 days- 14 yrs	M (2) F (3)	Seizures, diplopia, fatigue, loss of consciousness, hunger, inability to concentrate, sweating, clumsiness, drowsiness, weakness, incoordination of legs, slurred speech and hallucinations	0.8–24	NA/1–1.1
Boley et al. (1960) [30]	2	6 months; 10 months	M (1) F (1)	Seizures	3–6	NA/NA

*NA* Not Available, *F* Female, *M* Male

Here, we describe a case of hyperinsulinemic hypoglycemia due to insulinoma in a teenage girl. We also review other pediatric insulinoma cases published since 1960 and discuss the pitfalls of this potentially life threatening diagnosis in an adolescent patient.

## Case presentation

EM, a 15-year-old previously healthy Hispanic female, initially presented to an ED after she was found unconscious by her family at home in the afternoon during summer school vacation. Emergency medical services (EMS) described EM as “unresponsive and diaphoretic but without any tonic-clonic activity, eye rolling or incontinence”. Point of care (POC) glucose level was 29 mg/dl (1.6 mmol/L) and she was treated on site with 25 g Dextrose intravenously. The glucose level subsequently increased to 256 mg/dl (14.11 mmol/L) and EM woke up with amnesia. Reportedly, she had felt tired for the past 2 days, and was mostly sleeping, getting out of bed only to eat and urinate. The family reported that EM has not been using any medications, illicit drugs or alcohol. In the ED, her vital signs were stable and physical examination was only significant for acanthosis nigricans (AN). Her complete blood count, basal metabolic profile and urine drug screen were unremarkable. Given her negative history, serum sulfonylurea and blood alcohol levels were not done. She was observed for 5 h in the ED with multiple finger-stick glucose levels that were normal and was discharged at night with follow up requested in pediatric endocrine clinic in the morning. EM did not follow up with the endocrinology service due to insurance issues and inability to obtain a referral from her pediatrician whom she had not seen for over 3 years. She returned to the ED with hypoglycemia two days later. She was somnolent, minimally responsive and diaphoretic, with tremors in her lower limbs. EMS documented POC blood glucose of 19 mg/dl (1.05 mmol/L).

Further history revealed that EM had been more tired and shaky by the end of the school day for the previous 2 to 3 months. She had been having increased episodes of fatigue and sleepiness as well as jitteriness, tremors and sweating after school on days when she skipped both breakfast and lunch. Her symptoms resolved after a meal. EM had otherwise been well with a noticeable weight gain in the last 6 months, but without any incidence of illness, stress or hospitalization. Both EM and her mother denied any concerns at school or at home. They reported no glucose lowering medications at home, including insulin or sulfonylureas. EM also denied any alcohol or drug intake and thoughts of harming herself. Family history was negative for MEN1.

**Fig. 1:**
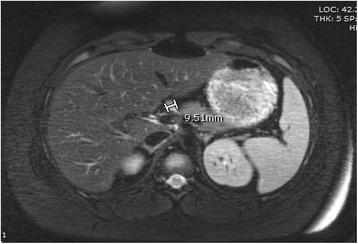
Axial T2 MRI image of the abdomen. The bar represents the 9.5 mm mass in the neck of the pancreas

Physical examination revealed a body mass index (BMI) of 32.8 kg/m^2^ (98th percentile) and mild AN of the neck. Renal and liver function tests and ammonia level were normal. Critical sample was obtained three times: all results revealed inappropriately high insulin levels without ketones and an inappropriate cortisol and growth hormone response to hypoglycemia (Table 2). A standard dose adrenocorticotropic hormone (ACTH) stimulation test showed a normal cortisol response (her baseline cortisol level of 6.6 mcg/dL (182.07 nmol/L) increased to 27 mcg/dL (744.83 nmol/L) following 250 mcg of cosyntropin administration. A 1 mg glucagon stimulation was performed: serum glucose levels increased from 39 mg/dl at baseline, after a 13-h fast to 64 mg/dL (3.53 mmol/L) and 77 mg/dL (4.24 mmol/L) at 10 min and 20 min respectively after glucagon administration and then dropped to 45 mg/dL (2.48 mmol/L) at 30 min. Abdominal magnetic resonance imaging (MRI) study showed a 1.0 cm mass in the pancreatic head (Fig. 1). To prevent hypoglycemia, she was administered dextrose-containing infusion, along with cornstarch supplementation. As insulinoma was suspected, she was referred to Children’s Hospital of Philadelphia for further care. A 1.2 cm × 1.2 cm mass was found on computed tomography (CT) scan of the abdomen with intravenous (IV) contrast and confirmed with microbubble contrast ultrasound study and 18 Fluoro-dopa positron emission tomography (18 F DOPA PET scan). EM subsequently underwent a partial pancreatectomy with removal of a 1.5 cm well-circumscribed insulinoma from the anterior-superior aspect of the pancreatic neck and diagnosis was confirmed by frozen section pathology. She tolerated the procedure without complications. She was able to maintain blood glucose levels without any extraneous support after the surgery. She remained euglycemic during a cure fast test, which was stopped after 41 h because of elevated ketones. She was discharged from the hospital and instructions for glucose monitoring were provided. At her follow up visit 3 months after removal of her tumor, she was clinically stable, lost weight (19 lb) and had no symptoms or evidence of hypoglycemia.

**Table 2:** Biochemical evaluation of critical samples

Test (unit)	Critical sample no. 1	Critical sample no. 2	Critical sample no. 3	Reference range (for euglycemia)
POC-glucose (mg/dL)	41	39	37	
Serum glucose (mg/dL)		48	44	60–99
Serum insulin (mcIU/mL)	63.2	14.8	28.5	2.6–24.9
C-peptide (ng/mL)	5.5	5.3	4.06	1.1–4.4
Cortisol (mcg/dL)	7.1	5.8	1.4	6.2–19.4 (am)
Betahydroxybutyrate (mmol/L)	0.29	0.27	<0.30	0.0–0.27
Serum Lactate (mmol/L)	0.7	1.2	1.0	0.5–2.2
Growth hormone (ng/mL)		2.43	2.78	0–3

## Discussion

We performed a thorough literature review and found less than one hundred pediatric insulinoma cases were reported since 1960 [8–30] (Table 1), of which three children had malignant insulinoma [9, 22, 26]. Presentation varied from subtle and non- specific to catastrophic events. The spectrum of symptoms included confusion, palpitation, sweating, tremors, hunger, irritability, dizziness, drowsiness, generalized weakness, abdominal pain, psychomotor slowing, syncope and seizures. Most of the episodes occurred during the morning hours, after exercise or fasting, and resolved after food or juice intake [9, 10, 14, 15, 17–19, 27]. Eighteen children experienced seizure activity [13, 14, 16, 18–21, 23–25, 27, 29, 30], four children were found in a comatose state [29, 30], three children had mental retardation as sequelae after surgery [11, 27, 30] and two children died [22, 30]. Duration of hypoglycemia symptoms before the diagnosis of insulinoma in children ranged from 1 month to 7 years, and averaged 10–13.4 months [14, 17]. The reasons behind the delayed diagnosis likely include not seeking medical attention due to symptom ambiguity [10], as well as failure of conventional imaging methods such as CT [10] and ultrasound [11] to localize the tumor. In our case, the family did not seek medical attention for at least 2–3 months because EM’s symptoms of increased fatigue, tremors, sweating, and somnolence followed skipped meals and resolved after eating, making her symptoms mimic hunger, until EM became unresponsive. This delay in diagnosis may be dangerous, since prolonged hypoglycemia can lead to seizures, mental status changes and sometimes death [30]. Halpin et al. recently reported a 15-year-old adolescent female with symptoms of fatigue, confusion, irritability and staring lasting for 5 months before she was found to have a 1.3 cm size insulin-secreting tumor [10]. This and our case emphasize that in the adolescent population, “normal” teenage behaviors can mask hypoglycemia symptoms, making the diagnosis more difficult in this age group.

EM’s physical examination indicated noticeable weight gain over the past few months and a BMI at the 98th percentile. Weight gain in insulinoma can be attributed to overeating to treat hypoglycemia symptoms, as patients discover that they feel better after food intake. There is also increased hunger due to release of catecholamines, occurring as a result of hypoglycemia, as low blood glucose levels stimulate the autonomic nervous system [31]. One study reported that 14% of the patients with insulinoma gained weight [32]; the incidence was even higher at 72% in another study [33]. The etiology of AN noted in our patient and also previously described in an obese 14-old-year male with insulinoma who initially presented with hypoglycemia and a 37 kg weight gain [16] is unclear and may be attributed to obesity-induced insulin resistance or increased insulin levels due to the insulinoma [16]. AN is thought to be caused by proliferation of fibroblasts and keratinocytes when excess insulin binds to insulin like growth factor-1 receptors [34]. This would lead one to believe that all cases of hyperinsulinism would be associated with AN; however, not all insulinomas present with AN [9, 10]. Why AN is present in some and not in others is not clear. What we know is that severe insulin resistance associated with obesity leads to compensatory hyperinsulinemia [35] and AN severity is proportional to increased insulin resistance [36]. Our patient’s AN could be a combined result of both obesity induced insulin resistance and hyperinsulinemia due to the insulinoma.

EM’s biochemical evaluation revealed inappropriately low cortisol and growth hormone levels in her critical samples (Table 2). These abnormal counter regulatory hormone responses to hypoglycemia in a patient with insulinoma have been previously documented [10, 16, 18, 37] and found to be due to a lower glycemic threshold for hormone release [37]. After tumor resection, the reversal in this pattern of cortisol response was noted (37). Inappropriately low counter-regulatory hormones for a low serum glucose level likely reflect the physiologic blunting seen due to chronic hypoglycemic stimuli and are most notable in patients who have diabetes mellitus, congenital hyperinsulinism and insulinoma [38]. In patients with diabetes mellitus and hyperinsulinism, hypoglycemia unawareness develops as recurrent iatrogenic hypoglycemia shifts the glycemic threshold for counter regulation and development of hypoglycemic symptoms to lower plasma glucose concentrations. The mechanisms underlying the development of hypoglycemia unawareness may be related to both altered central sensing of hypoglycemia and impaired coordination of responses to hypoglycemia [39]. Our patient’s delay in seeking medical care and discovery of hypoglycemia only after EM was found to be unresponsive with POC levels at 19 mg/dl (1.05 mmol/L) may be explained by this hypoglycemia unawareness.

The literature review suggests that imaging studies confirmed the clinically suspected diagnosis of insulinoma in 35 cases including ours. The tumors were first detected by MRI in 19 cases, by CT - in 12, by 18 F-DOPA PET - in 3, and ultrasound - in one case. Insulinoma in our patient was first detected by MRI, and later confirmed by ultrasound, CT and PET scans. Surgical removal of the tumor is the best treatment and considered in all cases (31), as it has very high cure rate [10, 17, 20]. Location of the tumor was reported in 47 cases including our case: tumors were found in the head of the pancreas in 14 cases, in the neck and the body - in 16 cases, and in the tail of the pancreas - in 17 cases. The size of resected insulinomas in the pediatric population ranged from 0.7 cm to 9.6 cm (9, 10, 11, 14, 15, 17, 18, 19) (Table 1) and averaged 1.14 cm (17) and 1.26 cm (14) in the noted studies. In our patient, 1.5 cm tumor was resected from the neck of the pancreas that promptly cured hypoglycemia symptoms.

Our case highlights the importance of careful evaluation of biochemical hypoglycemia. The diagnosis of insulinoma could be delayed because the symptoms are often non-specific.. Any patient presenting to the ED with documented Whipple’s triad, should be thoroughly investigated to rule out potentially life threatening exogenous and endogenous hyperinsulinemia and surreptitious use of oral hypoglycemic agents [40]. This patient’s initial presentation to the ED should have warranted a more detailed evaluation with an ultimate goal to establish the cause of hypoglycemia. Careful history (sometimes tracing back for a few months to years) including medication use and the presence of MEN1 in the family, physical examination and biochemical evaluation (serum glucose, insulin, C-peptide and urine and serum drug levels) are the initial steps in establishing a diagnosis and differentiating between the various causes. If hyperinsulinism is suspected, supervised fasting with a glucagon challenge test is the next step [1]. Our patient was discharged after symptomatic treatment, and before the cause of the hypoglycemia was established and was advised to see a pediatric endocrinologist for further investigation. This girl had not returned to her primary care doctor for surveillance physical exams for a few years and therefore could not promptly obtain a referral from him required by state health insurance in order to be evaluated by a pediatric endocrinologist. This situation delayed her access to specialized care and made the family return to the ED instead. Evaluation of her social situation, including health insurance at the initial ED visit may have changed disposition decision and prevented a second hypoglycemia event.

## Conclusion

In summary, this report describes a case of insulinoma in a previously healthy 15-year old female and highlights the challenges of the diagnosis. We reviewed the possible causes of delayed diagnosis: non-specific symptoms and hypoglycemia unawareness due to the diminished response of counter regulatory hormones. Although insulinoma is very rare in the pediatric population, delayed diagnosis can lead to prolonged and potentially dangerous hypoglycemia. The cause of hypoglycemia has to be established before a patient can be safely discharged home.

## Abbreviations

18F–DOPA PET: 18 Fluoro-dopa positron emission tomography
ACTH: Adrenocorticotropic hormone
AN: Acanthosis Nigricans
BMI: Body mass index
CT: Computed tomography
ED: Emergency department
EMS: Emergency medical services
MEN1: Multiple endocrine neoplasia type 1
MRI: Magnetic resonance imaging
POC: Point of care
